# Supplementary material for: Efficient multi-gene expression in cell-free droplet microreactors
Source: PLoS One. 2022 Mar 21;17(3):e0260420. doi: 10.1371/journal.pone.0260420 (PMC8936439; doi:10.1371/journal.pone.0260420)
Supplement: S1 File — (PDF) [file pone.0260420.s001.pdf]

Supplementary material:

Efficient multi-gene expression in cell-free droplet microreactors

Ana Maria Restrepo Sierra[1,2], Stefan Arold[1,3], and Raik Grünberg[1]

[1]Department of Biological and Environmental Sciences and Engineering, King Abdullah University of Science and Technology, Thuwal, Saudi Arabia
[2]Bionanoscience Department, Technische Universiteit Delft, Delft, The Netherlands
[3]Centre de Biochimie Structurale (CBS), CNRS, INSERM, Univ Montpellier, Montpellier, France

Contents

Supplementary Figures

S1 Calibration curves in bulk reactions A1
S2 Calibration curves in droplets A1
S3 Bxb1 purity A2
S4 Trade-off between binding efficiency and DNA loading levels. A2
S5 Comparison of different DNA coupling strategies for μl -scale expression. A3

Supplementary Tables

S1 DNA constructs A4
S2 DNA Oligonucleotides A5
S3 PCR products A5
S4 PCR program A A6
S5 PCR program B A6
S6 PCR program C A6
S7 Protein properties A7

Supplemental Figures

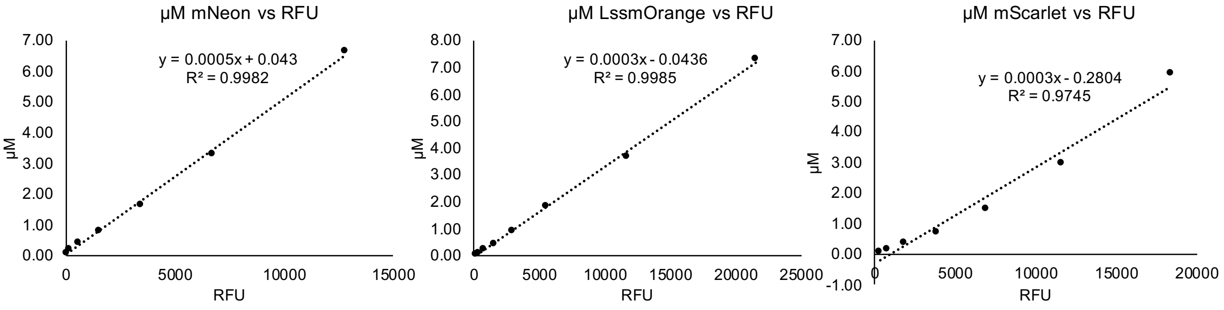

Figure S1: mNeonGreen, LSSmOrange, and mScarlet-I fluorescence calibration curves. Purified fluorescent proteins with defined concentration were measured at their respective peak excitation / emission wavelengths in 25 μl volumes.

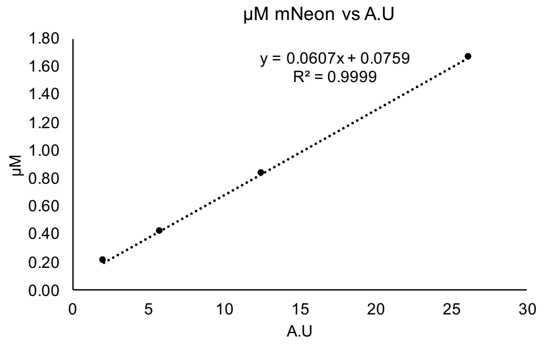

Figure S2: Purified mNeonGreen was encapsulated in droplets at defined concentrations and fluorescence was determined on a confocal microscope.

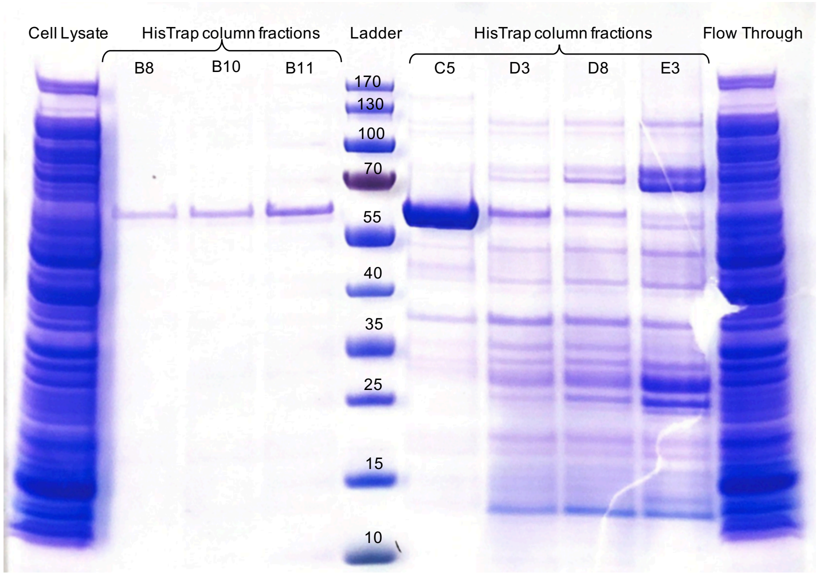

Figure S3: Bxb1 protein purification fractions after His-Trap column. Bxb1 MW: 58135 Da. Collected fractions for protein concentration were C2-C9

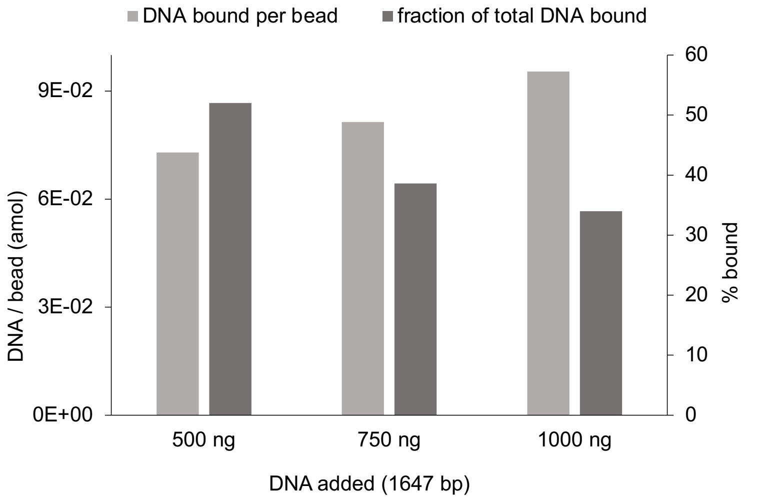

Figure S4: Binding efficiency of large DNA fragments (1600bp) (y-axis right) and DNA quantity bound per bead (y-axis left). The DNA binding reactions only differed on the DNA quantity added (between 500-1000 ng). The quantity of beads used remained constant ($3.5 \cdot 10^6$)

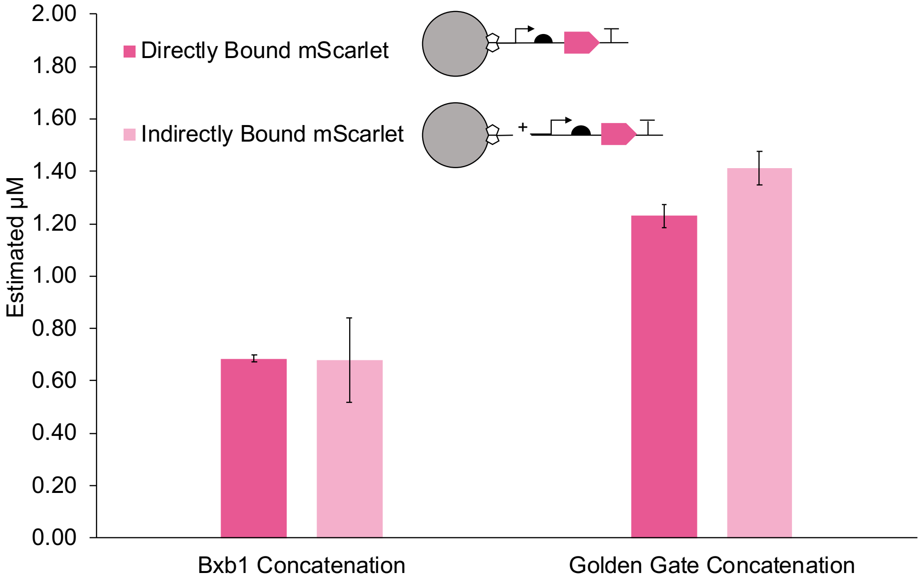

Figure S5: Comparison of DNA coupling strategies. Expression levels of mScarlet red fluorescent protein in 25 μl cell-free reactions were determined according to calibration curves in Figure S1. Error bars show the standard deviation from three replicates. PCR-amplified template DNA was either directly bound to beads (2000 ng per reaction with 7 μl beads) through biotinylation or the same amount of DNA was indirectly coupled by DNA assembly onto the same quantity of beads saturated with short immobilized oligonucleotides. Note that these are non-saturating DNA concentrations where also direct binding was expected to be efficient. Both approaches yield comparable expression levels indicating that a similar amount of DNA was immobilized. We noticed that DNA template quality mattered: independent of immobilization method, PCR-amplicons produced from non-clonal gBlock DNA tended to give lower expression levels (BxB1 assembly and control reaction on the left) than amplicons produced from the same gene cloned in a plasmid backbone (Golden Gate assembly and control on the right).

Supplemental Tables

Table S1: DNA constructs used in this study.

ID	type	composition	generated by
me0052	plasmid*	ALS-mScarletI-SpyTag-2Strep-pJEx411c	gene synthesis, ATUM
rg3032	plasmid*	FKBP-mNeon-WW-2Strep-pJEx411c	isothermal assembly
sb0215	plasmid*	2Strep-LssmOrange-SpyTag-pJEx411c	isothermal assembly
sb0201	plasmid*	Bxb1-His_{10}-pJEx411c	gene synthesis, Twist
rgf0046	dsDNA	random_sequence-*attP00*	gBlock, IDT
rgf0047	dsDNA*	*attB06*-FKBP-mNeon-WW-2Strep-*attP13*	gBlock, IDT
rgf0048	dsDNA*	*attB13*-2Strep-LssmOrange-SpyTag-*attP15*	gBlock, IDT
rgf0049	dsDNA*	*attB00*-ALS-mScarletI-SpyT-2Strep-*attP06*	gBlock, IDT

* expression cassettes include T7 promoter, lac operator, RBS insulator, RBS and T7 terminator.
Annotated DNA sequences of all constructs are available at: `[https://github.com/strubelab/dropletXpress]`

Table S2: DNA Oligonucleotides

ID	DNA Sequence
rgo119	5’ TEG-Biotin-ccttccgcgaaattaatacgactcac 3’
rgo120	5’ TEG-Biotin-cgatggtagtgtggggactcc 3’
rgo121	5’ cgatggtagtgtggggactcc 3’
aro023	5’ TEG-Biotin-tgcattcgtggatccgtatggaaccgcgagaccacggtt 3’
aro024	5’ aaccgtggtctcgcggttccatacggatccacgaatgca 3’
rgo144	5’ gcatttagaataaattttgtgtcgc 3’
rgo145	5’ gggtgtcgcccttagg 3’
aro013	5’ aacaatggtctccaccgcccttccgcgaaattaatacga 5’
aro014	5’ aactttggtctcgggcagcgatggtagtgtggggac 3’
aro015	5’ aacaatggtctcctgccccccttccgcgaaattaatacga 3’
aro016	5’ aactttggtctcgttgcgcgatggtagtgtggggac 3’
aro017	5’ aacaatggtctccgcaacccttccgcgaaattaatacga 3’
aro018	5’ aactttggtctcgtagtgcgatggtagtgtggggac 3’

Table S3: PCR fragments used in this study.

ID	template	composition	primers
PCR 1	me0052	ALS-mScarletI-SpyTag-2Strep	rgo119, rgo0120/121
PCR 2	me0052	ALS-mScarletI-SpyTag-2Strep	rgo119, aro014
PCR 3	rg3032	FKBP-mNeon-WW-2Strep	aro015, aro016
PCR 4	sb0215	2Strep-LssmOrange-SpyTag	aro017, aro018
PCR 5	rgf0049	*attB00*-ALS-mScarletI-SpyT-2Strep-*attP06*	rgo119, rgo145
PCR 6	rgf0047	*attB06*-FKBP-mNeon-WW-2Strep-*attP13*	rgo144, rgo145
PCR 7	rgf0048	*attB13*-2Strep-LssmOrange-SpyTag-*attP15*	rgo144, rgo145
PCR 8	me0052	ALS-mScarletI-SpyTag-2Strep	aro013, aro014
PCR 9	rgf0049	*attB00*-ALS-mScarletI-SpyT-2Strep-*attP06*	rgo144, rgo145
PCR 10	rgf0046	random_sequence-*attP00*	rgo119, rgo0121

All fragments include T7 promoter, lac operator, RBS insulator, RBS and T7 terminator. Annotated DNA sequences of all constructs are available at: [https://github.com/strubelab/dropletXpress]

Table S4: PCR program A: fragments bound directly on beads (PCR 1)

Step	Temperature	Time
Initial denaturation	98°C	30 sec
	98°C	10 sec
30 cycles	66°C	5-15 sec
	72°C	55 sec
Final extension	72°C	5 min

Table S5: PCR program B: Golden Gate assembly fragments (PCR 2, 3, 4)

Step	Temperature	Time
Initial denaturation	98°C	30 sec
	98°C	10 sec
5 cycles	63°C	15 sec
	72°C	55 sec
22 cycles	98°C	10 sec
	72°C	55 sec
Final extension	72°C	5 min

Table S6: PCR program C: Bxb1 recombination fragments (PCR 4, 5, 6)

Step	Temperature	Time
Initial denaturation	98°C	30 sec
	98°C	10 sec
25 cycles	60°C	20 sec
	72°C	55 sec
Final extension	72°C	5 min

Table S7: Proteins expressed in this study.

expressed from	composition	length (aa)	size (kD)	ϵ_{280} $(Mcm)^{-1}$	λ_{ex} (nm)	λ_{em} (nm)
me0052, rgf0049	ALS-mScarletI-SpyTag-2Strep	449	49.0	60,850	569	593
rg3032, rgf0047	FKBP-mNeon-WW-2Strep	447	48.9	77,810	506	517
sb0215, rgf0048	2Strep-LssmOrange-SpyTag	298	33.0	41,370	437	572
sb0201	Bxb1-His_{10}	515	58.1	86.400	-	-
